# Decreased Water Mobility Contributes To Increased α‐Synuclein Aggregation**

**DOI:** 10.1002/ange.202212063

**Published:** 2023-01-12

**Authors:** Amberley D. Stephens, Johanna Kölbel, Rani Moons, Chyi Wei Chung, Michael T. Ruggiero, Najet Mahmoudi, Talia A. Shmool, Thomas M. McCoy, Daniel Nietlispach, Alexander F. Routh, Frank Sobott, J. Axel Zeitler, Gabriele S. Kaminski Schierle

**Affiliations:** ^1^ Department of Chemical Engineering and Biotechnology University of Cambridge UK; ^2^ Department of Chemistry University of Antwerp Belgium; ^3^ Department of Chemistry University of Vermont USA; ^4^ ISIS STFC Rutherford Appleton Laboratory UK; ^5^ Department of Biochemistry University of Cambridge UK; ^6^ The Astbury Centre for Structural Molecular Biology University of Leeds UK

**Keywords:** Amyloid, Hydration Shell, Hydrogen Bond, Solvation Shell, Solvent

## Abstract

The solvation shell is essential for the folding and function of proteins, but how it contributes to protein misfolding and aggregation has still to be elucidated. We show that the mobility of solvation shell H_2_O molecules influences the aggregation rate of the amyloid protein α‐synuclein (αSyn), a protein associated with Parkinson's disease. When the mobility of H_2_O within the solvation shell is reduced by the presence of NaCl, αSyn aggregation rate increases. Conversely, in the presence CsI the mobility of the solvation shell is increased and αSyn aggregation is reduced. Changing the solvent from H_2_O to D_2_O leads to increased aggregation rates, indicating a solvent driven effect. We show the increased aggregation rate is not directly due to a change in the structural conformations of αSyn, it is also influenced by a reduction in both the H_2_O mobility and αSyn mobility. We propose that reduced mobility of αSyn contributes to increased aggregation by promoting intermolecular interactions.

## Graphical abstract

Ions can influence the mobility of water molecules in the solvation shell of a protein, which is important for folding and function. We show ions that decrease water mobility contribute to decreased protein mobility and subsequently to an increase in the self‐association and aggregation rate. Alteration in water mobility may occur in ageing cells where ion concentrations become dysregulated and therefore promote αSyn aggregation.

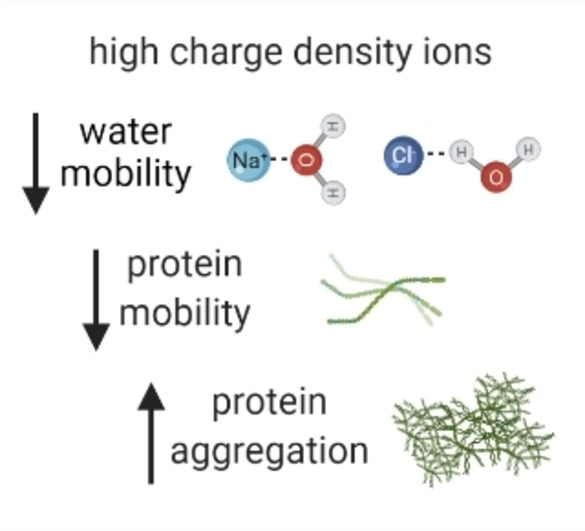

## Introduction

The majority of proteins cannot function without a solvation shell, and the mobility of this solvation layer affects rates of conformational change, catalysis and protein/DNA‐protein interactions.[^1^, ^2^, ^3^, ^4^] Solvent interaction is particularly pertinent for intrinsically disordered proteins (IDPs) which have large solvent accessible areas compared to globular proteins of a similar size.^[5]^ However, it is not currently clear what role the solvent plays in the misfolding and aggregation of proteins, particularly for IDPs like α‐synuclein (αSyn), whose aggregation is a hallmark of synucleinopathies, such as Parkinson's disease. Certainly, water molecules are expelled from the solvation shell for monomer‐monomer interactions, fibril elongation and fibril bundling to occur.[^6^, ^7^] Furthermore, it is well‐known that ions influence the hydrogen bond dynamics of water molecules, where small, high charge density ions lead to reduced water mobility, reduced diffusion and increased hydrogen bond lifetimes compared to in the presence of larger, low charge density ions.[^8^, ^9^, ^10^, ^11^, ^12^, ^13^] Despite this being recognised, the influence of salt ions on water mobility within differing cellular environments, and the subsequent impact this can have on protein misfolding, is currently not fully understood. Here, we show that the addition of NaCl, comprising two small, high charge density ions, and CsI, comprising two large, low charge density ions, can increase and decrease the aggregation rate of αSyn, respectively. These different salt solutions were chosen as it was previously shown that NaCl significantly reduced the hydrogen bond dynamics of water compared to CsI^[14]^ and also increased the aggregation propensity of αSyn.^[15]^ We reveal that water and αSyn mobility are inextricably linked and that increasing water mobility upon addition of CsI contributes to an increase in the protein mobility which reduces the propensity of αSyn to aggregate.

## Results and Discussion

### CsI Decreases αSyn Aggregation Rate Whereas NaCl and D_2_O Increase αSyn Aggregation Rate

**Figure 1:**
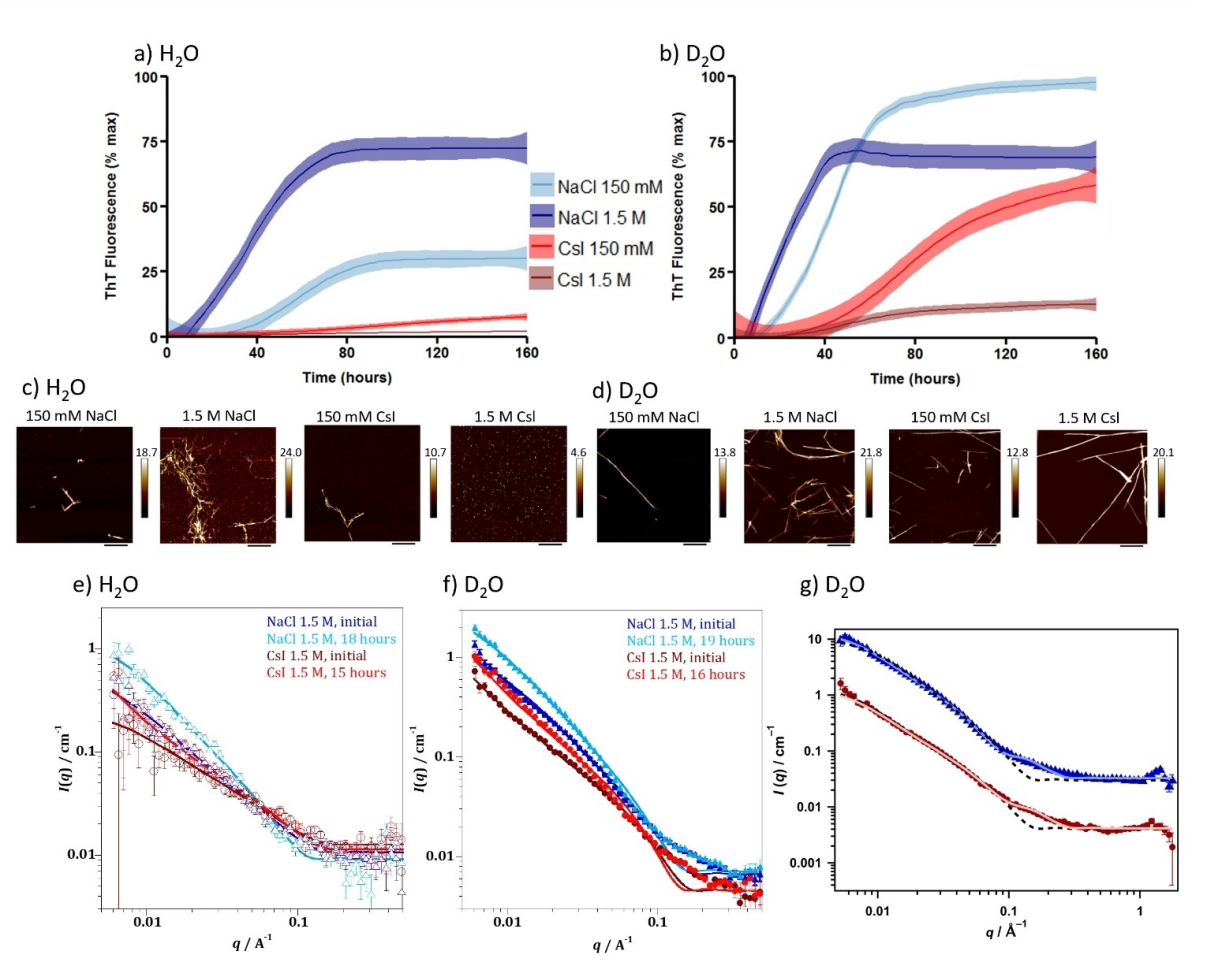
NaCl and CsI concentrations influence αSyn aggregation rate and morphology. αSyn aggregation kinetics were measured in the presence of a) H_2_O and b) D_2_O with 150 mM NaCl (light blue), 1.5 M NaCl (navy), 150 mM CsI (red), 1.5 M CsI (brown) and plotted as % maximum ThT fluorescence over time (Figure S1 displays individual plate repeats). Data represent three experiments with three or four wells per condition per experiment; error (shaded areas) represents rolling average of the SEM. After the ThT‐based assays, αSyn was incubated on freshly cleaved mica and representative images are shown for αSyn species formed in the presence of NaCl and CsI at 150 mM and 1.5 M in c) H_2_O and d) D_2_O. Scale bar=800 nm. For SANS measurements a high concentration (434 μM) of αSyn was used to ensure a sufficient number of scatter counts were attained. Model fits to the SANS data, using a flexible cylinder model, of αSyn in 1.5 M CsI and NaCl in e) H_2_O and f) D_2_O after initial mixing (NaCl—dark blue, CsI—dark red) and incubation for 15–19 hours (NaCl—light blue, CsI—light red). g) Raw SANS data of αSyn in 1.5 M NaCl, 19 hours (blue triangles) and αSyn in 1.5 M CsI, 16 hours (red circles) with fittings to a flexible cylinder with spheres (pale blue or red filled line) described more accurately the data than fitting to a flexible cylinders model only (dashed black line) using data from αSyn in 1.5 M salts in D_2_O. The NaCl (blue) is offset by a factor of 10 for clarity.

Aggregation rates of αSyn in the presence of NaCl and CsI were monitored using a fluorescence based aggregation assay which measures the fluorescence of Thioflavin‐T (ThT) as it intercalates into the backbone of β‐sheet containing fibrils.[^16^, ^17^] The sigmoidal kinetic curves, representative of a nucleation dependent aggregation reaction, show that aggregation of αSyn occurs faster in the presence of NaCl compared to CsI in H_2_O (Figure 1a, Figure S1). Furthermore, upon increasing the concentration of NaCl from 150 mM to 1.5 M, the αSyn aggregation rate increases further, as the time to form the first fibrillar structures decreases (referred to as the lag time (*t*_lag_)) and the elongation rate increases (slope of the exponential phase which indicates fibril elongation (*k*)) (Table 1). Conversely, aggregation of αSyn in the presence of CsI was slower at 1.5 M than at 150 mM concentrations and significantly slower compared to NaCl.

**Table 1:** Lag time (*t*_lag_), elongation rate (*k*), remaining monomer concentration determined by SEC and maximum fluorescence after performing ThT‐based kinetic assays.

Solvent	Salt concentration	*t*_lag_ [hours]	*k* [h^−1^]	Remaining Monomer [μM]	% max fluorescence at 160 hours
H_2_O	NaCl 150 mM	34.3±4.9	0.84±0.01	12.8±9.0	30.3±14.9
	NaCl 1.5 M	31.0±5.1	7.7±5.9	0	72.6±11.5
	CsI 150 mM	nd^[a]^	0.06±0.05	38.1±7.8	7.9±6.5
	CsI 1.5 M	nd^[a]^	0.01±0.01	35.9±16.6	2.1±1.0
D_2_O	NaCl 150 mM	23.8±4.0	2.5±0.6	2.0±3.0	98.1±0.9
	NaCl 1.5 M	19.0±2.6	6.1±2.5	0	69.0±15.3
	CsI 150 mM	39.3±0.9	0.65±0.36	11.7±2.7	59.1±25.1
	CsI 1.5 M	nd^[a]^	0.32±0.23	0	12.8±8.2

[a] nd not determined due to lack of detectable ThT fluorescence signal.

In order to further probe the influence of the solvent on αSyn aggregation, the same experiment is performed in a D_2_O containing buffer. We observe the same trends as for the H_2_O samples, i.e. the αSyn aggregation rate increases upon addition of NaCl, but decreases upon addition of CsI. However, when H_2_O is substituted for D_2_O, the αSyn aggregation rate is accelerated (Figure 1a,b, Table 1). We investigated the morphology of the resulting αSyn aggregates (Figure 1c,d, Figure S2) and the extent of aggregation by the quantity of remaining αSyn monomer after the kinetic assays (Table 1, Figure S3). The results mostly reflect the observed aggregation endpoints of the ThT‐based assays, but in the CsI containing samples oligomeric species are detected using atomic force microscopy (AFM) and size exclusion chromatography (SEC) (Figure 1c, Figure S3), species which do not lead to detectable ThT fluorescence.^[18]^

Since the formation of oligomeric species cannot be detected by ThT fluorescence, we used small angle neutron scattering (SANS) to evaluate size and structure differences of αSyn species formed in either NaCl or CsI containing buffers at early time points of the assay. Even at the initial time point, after αSyn was equilibrated in both salt solutions for 0.5 hrs in H_2_O and 1.5 hrs in D_2_O, SANS data show that αSyn species of larger sizes are already present in NaCl and D_2_O buffer, with an average radius of 19.5 Å. In contrast, αSyn species in CsI and D_2_O buffer had an average radius of 13.5 Å (Figure 1e–g, Figure S4, Tables S1 and S2). We use a flexible cylinder model and the Guinier‐Porod model to fit and analyse the SANS data (discussed in Supporting Information Note 1). After 16–19 hours, we show that there are four times more spherical monomeric αSyn species than cylindrical fibril‐like αSyn species in 1.5 M CsI and D_2_O, compared to three times more spherical monomeric αSyn in 1.5 M NaCl and D_2_O, which indicates that there are more fibrillar structures present in NaCl compared to CsI containing buffers (Table 2, Figure S4). The combined results suggest that CsI reduces the aggregation rate of αSyn compared to NaCl and that D_2_O increases the aggregation rate of αSyn compared to H_2_O.

**Table 2:** Parameters of fitting SANS data presented in Figure 1g. These results were obtained using a flexible cylinder and sphere model where sphere represents monomeric structures and cylinder fibrillar structures.

Solvent	Salt	*t* [hours]	Length [nm]	Kuhn length [nm]	Cylinder radius [Å]	Sphere radius [Å]	Cylinder scale factor	Sphere scale factor
D_2_O	NaCl	19	160	16.0	36.6	13.9	1.5×10^−4^	4.7×10^−4^
D_2_O	CsI	16	160	17.0	32.7	13.3	9.0×10^−5^	3.6×10^−4^

### The Mobility of Water Increases in the Bulk and in the Solvation Shell in the Presence of CsI Compared to NaCl

**Figure 2:**
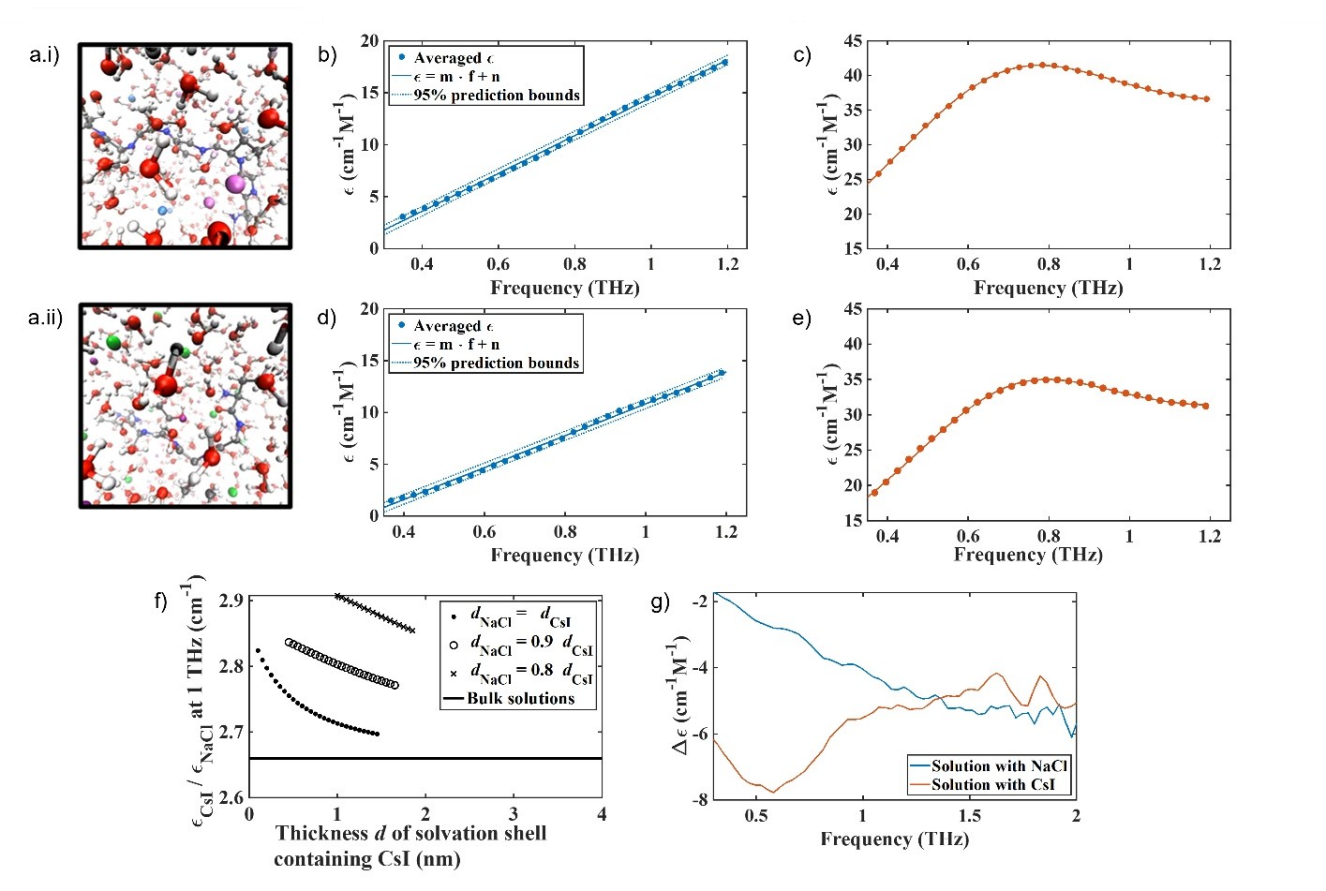
Addition of NaCl and CsI alter water mobility in the bulk and in the αSyn solvation shell. A snapshot of the AIMD simulations of the solvated αSyn_72‐78_ peptide in a 64 nm^3^ box after introduction and equilibration with 1.5 M salts. a.i) αSyn_72‐78_ peptide in 1.5 M CsI and a.ii) αSyn_72–78_ peptide in 1.5 M NaCl, Cs^+^ light purple, I^−^ light blue, Na^+^ dark purple, Cl^−^ green, O red, H white, C grey, N dark blue. The molar absorption coefficient measured with THz‐TDS for b) water and NaCl solutions, c) water and CsI solutions, d) water solutions of NaCl and αSyn, and e) water solutions of CsI and αSyn. b) and d) are fitted with a linear function and c) and e) with the sum of a power law and a Laurentzian to account for the spectral shape. Fitting parameters are found in Table S4. f) At 1 THz the water in the solvation shell surrounding αSyn containing CsI (ϵ_CsI_) absorbs more than the water shell containing NaCl (ϵ_NaCl_) and water of both solvation shells absorb more compared to bulk water only (black line at 2.66). The dependency of water absorption on the salt is plotted for several possible sizes *d* of the solvation shell, the ratios are shown in the box insert, allowing a smaller shell size *d*_NaCl_ in solutions containing NaCl. The same trend is apparent for all shell sizes. g) The absolute difference in molar absorption coefficient of water in the presence of the different salts and in the absence and presence of αSyn is shown for NaCl (blue) and CsI (orange). There is a smaller difference in the absorption of water in NaCl compared to water in CsI.

We first probed the mechanisms behind the differences in the aggregation rate of αSyn in the ionic solutions using ab initio molecular dynamics (AIMD) and classical MD simulations. We use a heptapeptide (TGVTAVA, residues 72–78) from the central region of αSyn, and a solvation environment similar to the experimental density and ionic concentrations studied. Note, we decided to use TGVTAVA as a model for αSyn, despite it being more hydrophobic than full length αSyn, as it was computationally more accessible than full length αSyn. We observe increased diffusion of the solvation shell, the bulk water and the peptide in the presence of CsI, with the diffusion of the peptide being most strongly affected. In the presence of water only and in NaCl, diffusion of the solvation shell, the bulk water and the peptide is decreased in comparison to in the presence of CsI (Figure 2a, Table S3), see Supporting Information Note 2 for further detail. The self‐diffusion constant of the heptapeptide is significantly elevated in the presence of CsI compared to water only as well as to NaCl models and indicate that the presence of these ions could have a great effect on both the water and the protein mobility in vitro. The rate of protein dimerisation is directly linked to the rate of protein reconfiguration, where slow reconfiguration permits dimerisation to occur, while fast reconfiguration reduces the likelihood of sustainable contacts that result in successful dimerisation.^[19]^

In order to study the effect of the different salts on water mobility in the bulk solution and in the solvation shell of the protein in vitro, and therefore to study the above observed computational effect experimentally on full length αSyn, we applied Terahertz time‐domain spectroscopy (THz‐TDS). THz‐TDS can be used as a highly sensitive probe to study water mobility in the liquid state as water absorbs more strongly in the THz domain than salt or protein. In particular, it can probe the complex interplay of molecular relaxation processes (dielectric relaxations and vibrational motions) that take place on timescales of ps to hundreds of fs by coupling to the infrared‐active dipoles of the molecular liquid.[^20^, ^21^] In this study, we used this method to analyse changes in the absorption of water in the presence and absence of the salts and αSyn protein. While the spectra of salts or water themselves may not show any spectral features in this range, the presence of salt ions in water changes the mobility and results in changes in the absorption coefficient measured. Indeed, similar broad spectral features have been reported in the terahertz spectra of ions in solution or organic solvents supporting our assumption that these vibrational modes could originate from hydrate like structures.[^22^, ^23^] In the absence of protein, we observe a larger overall increase of the absorption coefficient for water containing CsI than water containing NaCl (Figure 2b,c, Table S4). In line with previous results, samples containing αSyn protein led to a reduced absorption coefficient of water as the protein displaces the ions and water molecules, where water has a much stronger absorption than the protein due to the relative number of oscillators (Figure 2b–e, Table S4).[^24^, ^25^] Importantly, despite the absorption of water in NaCl being lower than in CsI in the absence and presence of protein (Figure 2b–e), upon addition of αSyn, the relative change in water absorption is greater in NaCl than CsI, showing that the water molecules are more influenced by the presence of NaCl than by the presence of CsI (Figure S5).

The absorption spectra of water in the presence of αSyn and the two salts were deconvoluted, and the absorption coefficient of the solvation shell surrounding the protein was calculated for a range of different supposed shell sizes and compared to the bulk absorption of water. In bulk, water containing CsI absorbs 2.66 times as much as water containing NaCl (flat black line, Figure 2f). When taking into account that the size of the solvation shell around the protein, which includes some ions, may depend on the salts, and especially the anions (calculated by AIMD in Figure S6), the water in the solvation shell of αSyn in a solution containing CsI is predicted to absorb between 2.7 and 2.9 times as much as the solvation shell in a solution containing NaCl in the largest mathematically possible solvation shell, i.e. in the case that solvation shells take up all available volume. It is found that the larger the assumed solvation shell, the larger its absorption coefficient, while still being lower than bulk water absorption. The absorption of water in the solvation shell is thus directly influenced by the interaction of the protein and salts and cannot be explained by the different absorption of hydrated salt ions only. The absolute difference in the absorption of water upon the addition of αSyn to a salt solution is overall smaller in the presence of NaCl than in the presence of CsI, showing that water mobility in the presence of NaCl is lower than in the presence of CsI (Figure 2g).

We investigated the influence of the salt on the water in the solvation shell, independent of the protein, as discussed further in Supporting Information Note 3, and observe that the absorption of water containing NaCl is lower than that of CsI (Figure S7, S8). Our THz‐TDS measurements have shown that adding the protein disturbs the interaction between water molecules and salt ions, and depends on the salt ion used. The changes in absorption measured by THz‐TDS are the combined effect of changes in the concentration of the molecular dipoles as well as their mobility. Increased water mobility results in stronger absorption, as does the increase in dipole concentration.

### αSyn Structure is Similar When Bound to Na^+^ and Cl^−^ as Cs^+^ and I^−^

**Figure 3:**
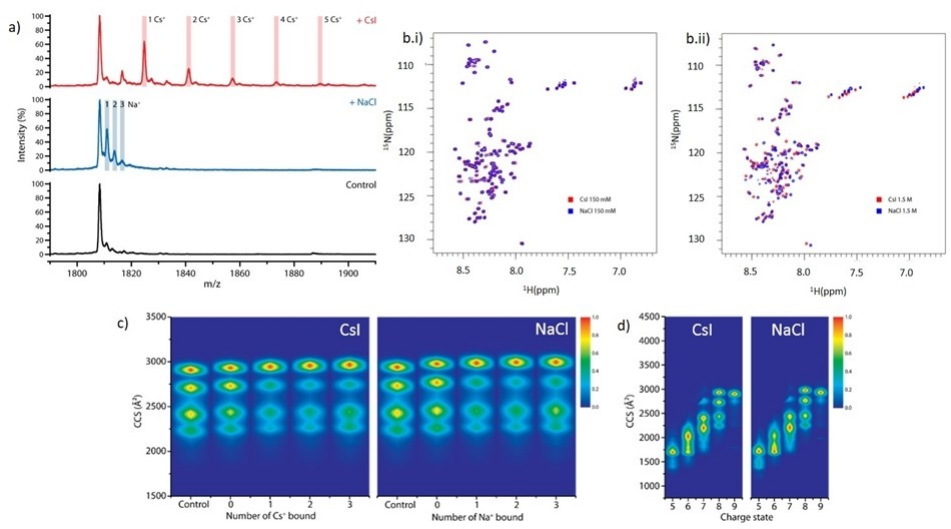
αSyn binds more Cs^+^ than Na^+^ which does not grossly affect αSyn conformation. The mass spectrum of a) native αSyn (Control, black) is shown in the 8+ charge state region, and in the presence of a 1 : 50 ratio (20 μM αSyn: 1 mM salt) we observe αSyn bound to three Na^+^ (+NaCl, blue) and to five Cs^+^ (+CsI, red). b) 2D ^1^H‐^15^N HSQC peak spectrum of αSyn containing (b.i) 150 mM CsI (red) in 5 % D_2_O, 95 % H_2_O (vol/vol) was overlaid with αSyn containing 150 mM NaCl (blue) in 5 % D_2_O, 95 % H_2_O (vol/vol). (b.ii) αSyn with 1.5 M NaCl (red) (vol/vol) was overlaid with αSyn containing 1.5 M CsI (blue). Gross shift perturbations are only observed across the protein sequence under very high (1.5 M) salt concentrations. c) Heat maps of the collisional cross section (CCS) Å^2^ of αSyn conformations detected for the 8+ charge state of αSyn without salt present and in the presence of 1 mM NaCl/CsI in a 1 : 50 protein:salt ratio and displayed in the absence of ions (Control), in the presence of ions but not binding (0), and as a function of the number of cations bound (1, 2, 3). d) Heat maps of the conformations of αSyn detected at different charge states (5+ to 9+) in the presence of 1 mM NaCl/CsI using a 1 : 50 protein:salt ratio.

We next investigated the possibility of structural differences of αSyn in the presence of NaCl and CsI, to determine whether the difference in aggregation rate could instead be due to a direct interaction of NaCl or CsI with αSyn. Native nano‐electrospray ionisation mass spectrometry (nano‐ESI‐MS) data show that αSyn binds a maximum of three Na^+^ and five Cs^+^ ions at a 1 : 50 ratio (20 μM αSyn: 1 mM salt) (Figure 3a) (data for 1 : 250 ratio is presented in the Figure S9 and discussed in Supporting Information Note 4). Binding of the counter anion I^−^ and Cl^−^ is not observed.

^15^N‐labelled αSyn was then measured by 2D ^15^N HSQC NMR spectroscopy to investigate structural changes in both 150 mM and 1.5 M CsI and NaCl solutions. The amide region of αSyn showed few chemical shift changes for residues in 150 mM salt solutions (Figure 3b.i), however large chemical shift changes were observed across all regions of αSyn in 1.5 M salt solutions (Figure 3b.ii). This suggests that there are no specific binding regions for the ions, but that salt binding at very high, non‐physiological concentrations may induce structural changes. However, the chemical shift changes observed by NMR reflect an ensemble average, weighted by the population of the conformation and aggregation states of αSyn in the salt solutions. As an IDP αSyn resides in many transient conformations, therefore we cannot clearly determine whether there are shifts in the distribution of αSyn conformations in the different salt solutions using this method.

We hence used nano‐ESI‐ion mobility‐MS (nano‐ESI‐IM–MS) to investigate potential changes to the distribution of αSyn conformations when bound to the salt ions. In the ion mobility experiment, the amount of gas‐phase collisions, and therefore the drift time, is directly related to the rotationally averaged extendedness of the protein ion.^[26]^ Using the 8+ charge state of αSyn in the absence of salt, we identified four main co‐existing conformations in the gas phase (Figure 3c), as previously reported.^[27]^ The choice of charge state represented is discussed in Supporting Information Note 5. The larger the collision cross sections (CCS), the more extended the protein structures. The binding of ions induced a shift favouring conformations with higher CCS values, but no further changes were observed with increased number of ions bound (Figure 3c, Figure S10). Overall, the αSyn conformational space did not extend or compact drastically, as has been observed for the binding of some small molecule drugs to αSyn,^[27]^ suggesting that the binding of these monovalent ions is non‐specific, similar to our observations by NMR and what we have observed with other monovalent ions.^[28]^ At lower charge states, slight differences in the intensity distribution of αSyn conformations in CsI compared to NaCl containing solutions could suggest that there are differences in the structural ensemble of αSyn, however these charge states may represent conformations that are influenced by the gas phase (Figure 3d). Both NMR and nano‐ESI‐IM–MS data suggest that there are no gross differences in the conformation of αSyn in the presence of CsI or NaCl. However, to fully determine different structures in these ensemble solutions a different technique may be required as the current resolution of these methods is not high enough.

### αSyn is More Mobile in CsI than in NaCl

**Figure 4:**
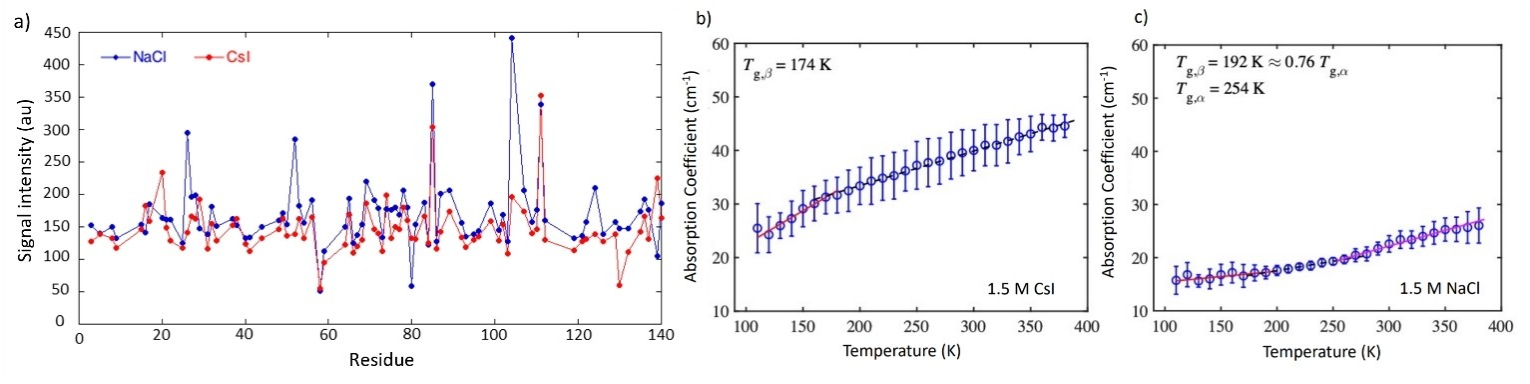
NMR and THz‐TDS show that αSyn is more mobile in CsI compared to NaCl. a) HSQC NMR spectroscopy was used to measure the intensity of 150 μM ^1^H and ^15^N‐labelled αSyn in 95 % H_2_O, 5 % D_2_O (vol/vol) containing 1.5 M CsI (red) and NaCl (blue). The signal intensity for αSyn is displayed for each salt with 86 % residue coverage. Each residue covered is represented by a dot. αSyn samples containing CsI had an overall lower intensity across most of the protein sequence. The mean terahertz absorption coefficient as a function of temperature at 1 THz is shown for b) αSyn and 1.5 M CsI and c) αSyn and 1.5 M NaCl. Red, black dashed and pink lines indicate the linear fits of the respective regions. *T*_g,*β*_ and *T*_g,*α*_ are defined as the intersect point between two linear fits. Error bars represent the standard deviation of 3 measurements.

We examined the effect of altered solvent mobility on the aggregation propensity of αSyn. MD simulations indicated that the altered water mobility, determined by the diffusion coefficient, in the solvation shell and the mobility of the αSyn_72‐78_ peptide backbone were inextricably linked, and the mobility of the αSyn_72‐78_ peptide chain in CsI solution was increased compared to in NaCl. We therefore examined the effects of the conformational rearrangement of αSyn in the two salts using ^15^N HSQC NMR spectroscopy and THz‐TDS. In these experiments, we used THz‐TDS to investigate the glass transition temperature of the αSyn protein in the presence of NaCl and CsI in a freeze‐dried form, thus in the absence of most water. For the NMR experiments we investigated the mobility of the protein in water containing NaCl and CsI. Both techniques showed that αSyn in NaCl differed in its mobility from αSyn in CsI. The altered mobility of ^15^N‐labelled αSyn led to reduced amide NMR signal intensities in CsI, which suggested that in the 1.5 M CsI solution the majority of residues of αSyn were sampling multiple conformations on timescales that contributed to additional signal broadening (Figure 4a). The apparent signal intensity losses caused by such exchange processes overtook any intensity gains anticipated from a faster rotational tumbling of αSyn in CsI solution. At the lower salt concentration (150 mM CsI) the difference was smaller but could still be observed (Figure S11). Furthermore, we observe that most of the protein sequence is influenced by the presence of NaCl and CsI, as there are no specific binding sites or regions for the ions present, which may lead to more localised intensity changes, and region‐specific peak shifts in the spectra, none of which have been observed (Figure 4a). The N‐terminal residues 1–20 were less influenced by the salt ions and were more similar in intensity.

Temperature ramping with THz‐TDS was used to investigate the onset of mobility by the temperature at glass transition points of solid state αSyn in CsI and NaCl (discussed further in Supporting Information Note 6). αSyn samples containing CsI become mobile at a lower temperature than αSyn samples containing NaCl and have a steeper gradient between *T*<*T*_g,β_, associated with local mobility (Figure 4b,c, Table S5). The THz‐TDS data are in agreement with our NMR data which show that αSyn samples containing CsI are more mobile and able to reconfigure than αSyn samples containing NaCl.

## Conclusion

To conclude, the influence of ions on the mobility of water has been well studied, yet the effect of water mobility on the propensity of proteins to misfold is still not elucidated and in particular, not in connection with IDPs and amyloid fibril formation. Here, we show that ions can influence the mobility of bulk water and water in the solvation shell, and the protein mobility, and that the dynamics of the surrounding solvent contributes to aggregation rates. The trend for increased αSyn aggregation rate in NaCl compared to CsI is observed in both H_2_O and in D_2_O, yet the aggregation rates of αSyn are faster in D_2_O. This suggests the solvent can influence αSyn aggregation rates and ions influence the solvent. The presence of deuterium bonds, which are stronger and shorter than hydrogen bonds,[^29^, ^30^] and the reduced diffusion constant of D_2_O compared to H_2_O,^[31]^ may increase the aggregation propensities of proteins.[^32^, ^33^, ^34^, ^35^, ^36^] We directly observe that the presence of CsI leads to increased water mobility, both in bulk and in the protein solvation shell, in comparison to NaCl. An increase in absorption, as measured with THz‐TDS, directly relates to an increased change in dipole moment and therefore ion and protein mobility which are inextricably linked to the mobility of surrounding water molecules.

Although direct ion binding has been proposed to influence αSyn aggregation rates, the ion binding strength does not correlate with aggregation rates observed,^[37]^ suggesting that the Hofmeister series may not be the only explanation for why these ions either decrease or increase αSyn aggregation kinetics. Furthermore, we can exclude the Debye–Hückel effect as both NaCl and CsI are monovalent; if such a charge screening effect was dominant, a similar effect on the aggregation kinetics of αSyn should have been observed. Structural alterations to the dynamic ensemble of αSyn conformations by NaCl and CsI, which may favour aggregation prone conformations, cannot be ruled out. Although we observed no gross differences in the structures of αSyn by NMR and MS in the presence of NaCl and CsI, these techniques may not be sensitive enough on the timescale needed to identify differences in transient dynamic interactions within the monomer structures in solution. Yet, these dynamic interactions govern whether a protein remains monomeric or misfolds into conformations that can aggregate. The surrounding solvent dictates the time scale for forming and maintaining these conformations.

Although solvent motions are on the fs to ps timescale and the rate of conformational changes in proteins occur on the ns‐ms timescale, the solvent mobility still has a knock‐on effect to the motions of the protein. It has been well studied that an increase in mean square displacement of hydrated proteins occurs at ≈220 K, which is absent in dehydrated proteins, and is related to rotational dynamics of water and the side chain motions of the amino acid chain,[^38^, ^39^] where methyl group rotations occur on the ps timescale and solvent dependent amino acid motions and localised diffusion occur on the ps‐ns timescale.^[40]^ As an IDP, αSyn undergoes more hydrogen bond interactions with the surrounding solvent compared to a folded protein of similar chain length, making αSyn highly sensitive to the surrounding solvent environment. The coupling of water motions, the presence of ions and protein dynamics are protein specific due to differences in charge, hydrophobicity, extent of solvent exposure and degree of surface roughness.^[41]^ This is also apparent within the different regions of αSyn, where reorientation times of water differ dependent on the amino acid composition. Reorientation dynamics of water at the hydrophobic, aggregation prone region were much slower than those observed at either of the charged termini.^[42]^

NMR and MD simulations have shown that the hydrogen bond lifetimes of the surrounding water molecules have subsequent effects on the ps motions of segments of the disordered region of a Sendai virus protein, in particular effecting the motions of sidechains and the twisting of the backbone.^[43]^ For many proteins, when the reconfiguration rates of the protein backbone are retarded, this can lead to aggregation.[^19^, ^42^, ^44^, ^45^, ^46^, ^47^, ^48^] For protein association and aggregation to occur, the proteins must firstly be in an aggregation prone conformation, and secondly must be stable for long enough for interactions to occur. When the surrounding solvent is reduced in its motion, this not only influences protein motions, but may also increase intermolecular interactions; further study will be needed to determine if increased hydrogen bond lifetimes between protein and solvent by the presence of ions or osmolytes alters the likelihood of its associations. We extend the concept for αSyn that slowing down the motion of the solvent can reduce ps motions of the protein, thereby stabilising the protein for longer in a more aggregation‐prone, but still monomeric, conformation (as shown recently;^[49]^). This thereby increases the likelihood of the occurrence of more ns‐ms structural changes as highlighted by an increase in the formation of αSyn fibrils in the presence of NaCl.

**Figure 5:**
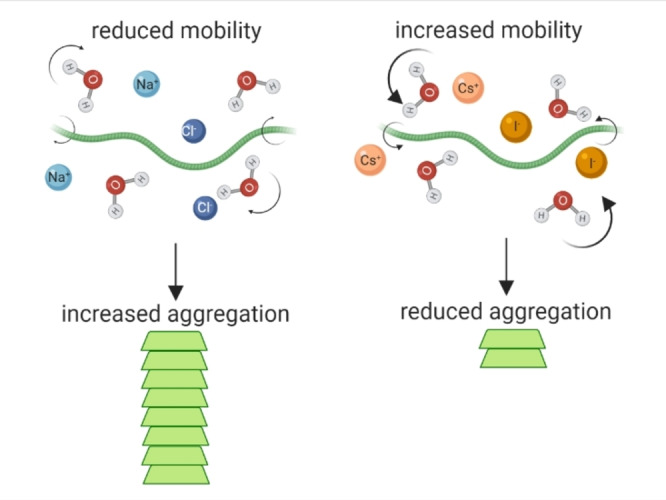
Aggregation kinetics of αSyn is influenced by water and protein mobility which is strongly affected by the presence of ions. Presence of Na^+^ (light blue) and Cl^−^ (dark blue) lead to reduced mobility of water (H_2_O) and αSyn monomers (green protein), allowing the formation of stable intermolecular bonds between two adjacent monomers which can lead to aggregation into αSyn amyloid fibrils (stacked green protein). Presence of Cs^+^ (light orange) and I^−^ (dark orange) lead to increased mobility of water and αSyn monomer, which decreases the likelihood of two monomers being stable enough to permit intermolecular interactions and thus results in reduced aggregation. Created with BioRender.com.

While many factors affect aggregation rate and aggregation propensity of αSyn and other amyloidogenic proteins, including protein concentration, amino acid composition, temperature, pH, molecular crowding, osmolytes and lipids, our data also supports a mechanism whereby the pathway to oligomerisation and aggregation is further influenced by the intramolecular diffusion rate of the protein, which we show is effected by the mobility of, and intermolecular interactions with, the surrounding water, which in turn is modulated by the ions present (Figure 5). Furthermore, the presence of ions and osmolytes in cells, which differ dependent on the cell type, can influence water hydrogen bonding, protein‐water bonds and their lifetimes.^[50]^ These data may have important implications for αSyn localised within certain environments either inside or outside of a cell, where ion concentrations can differ greatly.[^51^, ^52^] The presence of ions during the formation of the yeast prion protein oligomers, but not during elongation, can influence fibril polymorphism and is directly linked to pathology.[^53^, ^54^] Therefore, interesting questions arise regarding cell specific or age dependent accumulation of certain ions or metabolites in the intracellular aqueous environment that could alter water mobility and influence αSyn aggregation, strain polymorphism and disease outcome.

## Conflict of interest

The authors declare no conflict of interest.

1

## Supporting information

As a service to our authors and readers, this journal provides supporting information supplied by the authors. Such materials are peer reviewed and may be re‐organized for online delivery, but are not copy‐edited or typeset. Technical support issues arising from supporting information (other than missing files) should be addressed to the authors.

Supporting Information

## Data Availability

The data that support the findings of this study are openly available in University of Cambridge Repository reference number 63587.
